# The *European Journal of Neurology*: 2025 and Beyond

**DOI:** 10.1111/ene.70170

**Published:** 2025-06-19

**Authors:** Claudia Sommer

**Affiliations:** ^1^ Universitatsklinikum Würzburg Würzburg Germany

It is my great honor and privilege to succeed Prof. Didier Leys as the Editor‐in‐Chief of the EAN flagship journal, the *European Journal of Neurology* in July 2025. Under Didier Leys' exemplary leadership, I had the opportunity to serve as part of the associate editors' team since 2020. I am deeply grateful for his mentorship and guidance, which have been invaluable in shaping my editorial journey. I would also like to express my heartfelt gratitude to my esteemed colleagues who have served as associate editors alongside Didier and me—listed alphabetically: Barbara Borroni (Brescia, Italy), Andrew Chan (Bern, Switzerland), Mark Edwards (London, UK), Alessandro Tessitore (Naples, Italy), Kristl Vonck (Ghent, Belgium)—and to the outstanding guest editors who have played a crucial role in bringing special issues to life. As we transition, some associate editors will continue, while others will move on due to professional commitments. I am pleased to announce that Mark Edwards has accepted the position of Deputy Editor, starting in July 2025, and that Andrew Chan and Kristl Vonck will stay on as associate editors. The new associate editors will be Federica Agosta (Milan, Italy), Janika Kõrv (Tartu, Estonia) and Stojan Peric (Belgrade, Serbia). I am very much looking forward to working with this outstanding team.

Since 2020, the *European Journal of Neurology* has undergone substantial changes, including the adoption of an open‐access model to align with the EAN's commitment to open science and improve accessibility to advancements in clinical research. We also introduced an annual award for the best paper of the year, which, starting in 2025, will include a monetary prize, made possible by the generous donation of Jes Olesen. Furthermore, the Journal has strengthened its outreach by appointing social media editor Burak Kilboz, significantly increasing its presence across various platforms.

With a diverse selection of original research, review articles, guidelines, and opinion pieces, the *European Journal of Neurology* remains at the forefront of European neurological science. Beyond publishing, it has embraced innovative formats such as interviews, videos, podcasts, and even launched its own symposium at the EAN's annual congress. My goal is to build on these achievements and further enhance the Journal's role in open science. This includes promoting transparency in data sharing, educating authors on best practices in open science, and working closely with the EAN and our publisher Wiley to support these efforts.

*The European Journal of Neurology* serves European clinical scientists and a global audience seeking guidance, comparisons, or insights into European clinical neurology. It encompasses the full spectrum of clinical and translational research, including epidemiology, preventive medicine, and health economics, provided the content remains accessible to general neurologists. Contributions on both common and rare diseases are equally encouraged.

As the official journal of the EAN, it stands as the organization's flagship publication, embodying its vision and values. To maximize its impact, the *European Journal of Neurology* must integrate seamlessly with the EAN's other platforms, such as EAN News and EAN Campus. A key priority for me is to actively engage neurologists in training, particularly RRFS members. Young neurologists, who frequently contribute to the Journal, deserve a dedicated platform to showcase their achievements. I plan to introduce a section highlighting their work, featuring commentaries on published articles or emerging developments, and illustrating how the Journal has influenced their careers. In collaboration with the EAN, we will provide training opportunities to equip young neurologists with essential reviewing skills. We will follow up on these courses with hands‐on experience for early‐career reviewers and work to increase their representation on the Editorial Board.

The Journal's submissions reflect a truly global reach, attracting contributions not only from Europe but also from Asia, North and South America, Australia, and North Africa. This diversity is mirrored in our Editorial Board. Some of our Editorial Board members will have finished their term by the end of 2025, and I would already like to thank them for their engagement for our journal in the past years. We will recruit new Editorial Board members later this year. If you are interested, contact me, but please be aware that we aim to keep a good balance regarding regions, gender, subspecialty, and stage of career. Editorial Board member tasks are, among others, to serve as reviewers in contentious cases, to make suggestions for special issues and commissioned review papers, and to review journal performance metrics, such as submission statistics, geographical distribution, and strategies to increase diversity.

To uphold the Journal's standards and provide authors with constructive feedback, we rely on an exceptional pool of reviewers. Peer review is a cornerstone of academic publishing, yet it is often challenging to secure the time and expertise of busy clinicians and scientists. To address this, we will continue to ensure that only high‐quality manuscripts deserving of consideration are sent for peer review, thereby respecting reviewers' time and effort. Furthermore, we will expand our incentives for frequent and exemplary reviewers and strengthen communication with them to maintain a robust peer review process.

Ultimately, the *European Journal of Neurology* thrives because of its contributors. I encourage EAN members to submit their highest‐quality clinical or clinically relevant basic research. With our open‐access model, your work will reach a global audience and benefit from increased visibility through our active social media channels. Additionally, the best manuscript of the year will be recognized at the EAN Congress and awarded a prize. Senior authors are also invited to contribute review articles, sharing their expertise to educate our readers and further enrich the Journal's content.

Together, we can ensure that the *European Journal of Neurology* remains a leading platform for clinical science, driving knowledge forward and fostering global collaboration.

## Author Contributions

**Claudia Sommer:** conceptualization, writing – original draft, writing – review and editing.

## Conflicts of Interest

The author declares no conflicts of interest.

## Data Availability

Data sharing not applicable to this article as no datasets were generated or analysed during the current study.

